# Magneto-Fluorescent Mesoporous Nanocarriers for the Dual-Delivery of Ofloxacin and Doxorubicin to Tackle Opportunistic Bacterial Infections in Colorectal Cancer

**DOI:** 10.3390/ijms232012287

**Published:** 2022-10-14

**Authors:** Gonçalo A. Marcelo, Joana Galhano, Tiago T. Robalo, Maria Margarida Cruz, María D. Marcos, Ramón Martínez-Máñez, Maria Paula Duarte, José Luis Capelo-Martínez, Carlos Lodeiro, Elisabete Oliveira

**Affiliations:** 1 BIOSCOPE Group, LAQV@REQUIMTE, Chemistry Department, FCT NOVA, 2829-516 Caparica, Portugal; 2 BioISI-Instituto de Biosistemas e Ciências Integrativas, Faculdade de Ciências, Universidade de Lisboa, 1749-016 Lisboa, Portugal; 3 Instituto Interuniversitario de Investigación de Reconocimiento Molecular y Desarrollo Tecnológico, Universitat Politècnica de València, Universitat de València, 46022 Valencia, Spain; 4 Unidad Mixta UPV-CIPF de Investigación en Mecanismos de Enfermedades y Nanomedicina, València, Centro de Investigación Príncipe Felipe, Universitat Politècnica de València, 46100 Valencia, Spain; 5 CIBER de Bioingenieria, Biomateriales y Nanomedicina (CIBER-BBN), 28029 Madrid, Spain; 6 Chemistry Department, FCT NOVA, 2829-516 Caparica, Portugal; 7 PROTEOMASS Scientific Society, Rua dos Inventores, Caparica Campus, 2829-182 Caparica, Portugal

**Keywords:** magneto-fluorescence, mesoporous nanoparticles, drug delivery, hyperthermia, antimicrobial, opportunistic cancer-related bacteria

## Abstract

Cancer-related opportunistic bacterial infections are one major barrier for successful clinical therapies, often correlated to the production of genotoxic factors and higher cancer incidence. Although dual anticancer and antimicrobial therapies are a growing therapeutic fashion, they still fall short when it comes to specific delivery and local action in in vivo systems. Nanoparticles are seen as potential therapeutic vectors, be it by means of their intrinsic antibacterial properties and effective delivery capacity, or by means of their repeatedly reported modulation and maneuverability. Herein we report on the production of a biocompatible, antimicrobial magneto-fluorescent nanosystem (NANO3) for the delivery of a dual doxorubicin–ofloxacin formulation against cancer-related bacterial infections. The drug delivery capacity, rendered by its mesoporous silica matrix, is confirmed by the high loading capacity and stimuli-driven release of both drugs, with preference for tumor-like acidic media. The pH-dependent emission of its surface fluorescent SiQDs, provides an insight into NANO3 surface behavior and pore availability, with the SiQDs working as pore gates. Hyperthermia induces heat generation to febrile temperatures, doubling drug release. NANO3-loaded systems demonstrate significant antimicrobial activity, specifically after the application of hyperthermia conditions. NANO3 structure and antimicrobial properties confirm their potential use in a future dual anticancer and antimicrobial therapeutical vector, due to their drug loading capacity and their surface availability for further modification with bioactive, targeting species.

## 1. Introduction

Cancer-associated bacterial infections and inflammations have been pointed to as one of the main causes of low treatment success rates and treatment-associated complications [1,2,3]. Bacterial infections are particularly damaging in gastric and colorectal cancer (CRC) cases, with strains like *Helicobacter pylori* and *Escherichia coli* being linked to cancer invasiveness and progression [2,4,5,6,7]. This, allied to the increased occurrence of antimicrobial resistant bacteria, poses a severe threat to current cancer therapy successful rates, but, most importantly, to patients’ health and survival [8,9,10].

Strategies to fight bacterial growth and surpass existing resistances are a current hot topic, with several worldwide research groups and institutions developing distinct approaches. These distinct approaches include the production of new antibiotics [11], the use of naturally occurring molecules of animal or plant origin [12,13], the combinatory use of conventional antibiotics and adjuvants [14,15], or the use of engineered nanomaterials [16,17]. In particular, nanomaterials have gained special attention throughout the last decades, due, particularly, to their intrinsic properties, tunability and easier manipulation. For instance, silver and copper nanoparticles, among others, are examples of nanoparticle systems whose constituents have antimicrobial properties [18,19,20]. Iron oxide nanoparticles, for example, have been of great interest as they not only bring greater control and maneuverability over therapies, due to their magnetic properties, but have also been reported to have antimicrobial and anti-biofilm properties [21,22,23]. Moreover, the use of magnetic iron oxide nanoparticles as approved contrasting agents, heat generating and delivery probes in several diagnostic and therapeutic approaches, particularly in cases of cancer, demonstrates its important biocompatibility and safe use in humans [24,25,26]. Non-antimicrobial nanoparticles have also been the stage for the production of antimicrobial solutions, by conjugating an initial non-toxic matrix with conventional bacteriostatic or bactericidal agents. This conjugation has been reported to produce synergic therapeutic effects between its constituents, be it through joint action, or by the masking and effective delivery of the antimicrobial agent, by the second material, to its target [27,28,29]. A wide variety of non-toxic matrices has been reported in the literature, such as gold and mesoporous silica nanoparticles, whose surfaces can not only be easily functionalized, but also work as good anchoring sites for bioactive molecules [28,30,31,32,33]. Moreover, additional functionalization of nanoparticles’ surfaces [34,35] and nanoparticles’ combination [36,37] render them with extra properties, from controlled and targeted drug delivery to stimuli responsive behavior, long-term blood circulation, higher cellular take-up, magnetic- or light-induced heat generation and fluorescence, to name a few, that can be used to improve their antimicrobial properties.

All considered, the production of adapted nanotherapies, capable of tackling both cancer and bacterial infections is, thus, of utter relevance [38]. This is supported by the recent, yet scarce, reports on the use of antibiotics as anticancer targets [39] and of anticancer drugs as potential antimicrobial agents [40], as well as their synergic effects in combined therapy [41]. In a first step to address this, in our group we have recently reported the use of mesoporous silica nanoparticles for the combined therapy against several cancer-related bacterial strains, using doxorubicin, epirubicin and ofloxacin as model anticancer and antimicrobial drugs [27]. The combination of these two classes of drugs allowed us to produce an efficient nano-based therapy, with better activity than their constituents alone and the capacity to target resistant strains, such as methicillin-resistant *Staphylococcus aureus* (MRSA).

In an attempt to improve the system, and prepare it for further application against cancer and related bacterial infections, in this work we report on the synthesis of a magneto-fluorescent mesoporous nanosystem for the combinatory delivery of doxorubicin and ofloxacin, as model anticancer drug and antibiotic. With the produced nanosystem, comprised of a superparamagnetic iron oxide core, a mesoporous silica matrix and fluorescent silicon quantum dots (SiQDs), we seek to bring together the hyperthermia properties of the magnetic core [22,26], the cargo capacity of the mesoporous silica and the trackable fluorescence of SiQDs [42], while using sustainable, biocompatible, and biodegradable materials. The antimicrobial activity is tested against a set of common bacterial strains, usually associated with cancer, to validate their application in a combined therapy.

## 2. Results and Discussion

### 2.1. Characterization of NANO2 and NANO3 Systems

**Figure 1:**
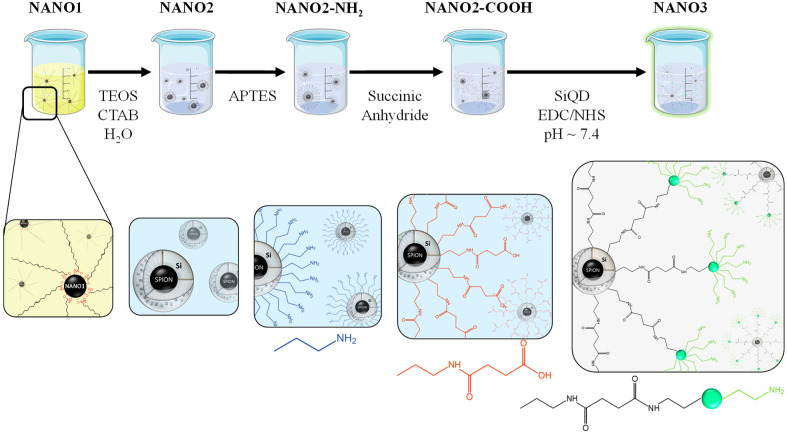
Sequential representation of the step-by-step synthesis of NANO3, from the formation of NANO1, and dispersion in CHCl_3_, to the growth of a mesoporous silica shell (NANO2) in water, further surface modifications (NANO2-NH_2_ and NANO2-COOH) and SiQD anchoring via amidation. Representations not at real scale.

Magneto-fluorescent mesoporous nanoparticles were successfully obtained in a step-by-step synthetic approach, from NANO1 to NANO3, using iron oxide, mesoporous silica, and silicon as the main building block reagents (Figure 1).

The initial production of the NANO1 magnetic Fe_3_O_4_ cores followed a typical co-precipitation method of Fe^2+^ and Fe^3+^ salts, in the presence of oleic acid as surface stabilizer and modulator [43]. NANO1 were obtained as homogeneous and small sized spherical nanoparticles (Figure S1), with an average diameter of 11.73 ± 1.47 nm, typical of ultra-small superparamagnetic nanoparticles (SPIONs) [36]. The superparamagnetism of NANO1 arises from its small dimensions that ensure the existence of a single reverse spinel phase iron oxide crystalline structure and, thus, a homogeneous and fast response to an external magnetic field [44]. This was confirmed even after the grafting of the mesoporous silica shell, by XRD and TEM analysis of NANO2, that showed not only a typical SiO_2_ XRD pattern and Fe_3_O_4_ 2θ peaks at 30.26°, 35.60°, 43.36°, 53.72°, 57.16° and 62.65° (*hkl* planes: <220>, <311>, <400>, <422>, <511> and <400>, respectively) [45], but also single oriented core crystalline structures, with calculated interplanar distances matching those obtained by XRD (Figure S2).

The growth of the mesoporous silica shell onto NANO1’s surface was obtained by an initial surface co-stabilization with oleic acid and CTAB, where the alkyl chains of OA and CTA+ re interdigitated. The shifting of NANO1 to the aqueous phase was then followed by the well-established Stöber method for the synthesis of MCM-41. Here, TEOS was used as the silica source, ethylene glycol as the surface stabilizer and NH_4_OH as the reducing and morphological agent. The mesoporosity of the matrix was ensured by the presence of CTAB, that worked as a cationic templating surfactant. NANO2 were obtained in the form of a brown, magnetic powder and in a concentration of ca. 2.0 × 10^13^ nanoparticles/mg. Further surface modifications, first with APTES and then with succinic anhydride to yield NANO2-COOH, and with SiQDs (via EDC/NHS cross-linking) to yield NANO3, were continuously followed by DLS measurements of their hydrodynamic diameter (HD) and zeta potential (ζ) in water (Figure S3). The sequential surface modifications were complemented by appropriate changes in surface charge, with bare NANO2 having an initial net negative charge (−27.7 ± 0.4 mV), typical of non-templated mesoporous silicas [46], which changed into +50.8 ± 0.7 mV after the grafting of APTES and its -NH_2_ terminal groups, and later reverted to −39.2 ± 1.3 mV with the addition of succinic anhydride, that underwent a ring opening amidation reaction to form terminal succinamic acid moieties. The successful addition of SiQDs to NANO2-COOH, by EDC/NHS mediated amidation with amine surface groups of the former, was translated by an inversion of ζ to +12.4 ± 1.3 mV in NANO3. All registered changes in ζ influenced the final HD of each NANO system, with NANO2-NH_2_ and NANO2-COOH having the highest surface charges corresponding to the smallest HDs (203.3 ± 2.9 nm and 115.5 ± 4.7 nm, respectively) and, thus, increased dispersibility and stability in water. The addition of the nanometric SiQDs to its surface, as indicated by NANO3’s ζ, destabilized the system and resulted in a large HD of 1076.0 ± 159.9 nm. This was a consequence of the hair-like distribution of the surface chains of NANO3, whose fluctuations made it non-homogeneous over time, and the weak surface charge that arose from the low protonation of surface -NH_2_ moieties from the anchored SiQDs (pKa _immobilized-APTES_ = 7.6 [47,48]) in the aqueous medium (pH = 6.8). The overall positive charge of the final NANO3 was postulated as a potential asset in antimicrobial applications, as recent studies have pointed to positively charged particles as being the most effective against bacterial strains [49,50]. SiQD concentration during the cross-linking reaction was adjusted to ensure NANO2-COOH surface saturation and to avoid interparticle cross-linkage.

Surface modifications were also confirmed by FTIR spectroscopy, with carboxylic acid (strong-broad O-H stretch, 3000 cm^−1^; strong C=O stretch, 1715 cm^−1^; medium O-H bend, 1400 cm^−1^; strong C-O stretch, 1320 cm^−1^) and secondary amide peaks (weak C=O stretch, 1635 cm^−1^; medium N-H bend, 1545 cm^−1^) emerging in the NANO2-COOH spectrum, and later turning into solely secondary amide (weak C=O stretch, 1635 cm^−1^; medium N-H bend, 1545 cm^−1^) and primary amine (weak-broad N-H stretch, 3350 cm^−1^) signals in NANO3 (Figure S4). Moreover, successful template removal was translated by the absence of the typical strong C-H stretching between 2900 and 3000 cm^−1^, in the NANO2 spectrum. Conversely, the increase of the same signals in NANO2-COOH and NANO3 confirmed the presence of the aliphatic linking chains at their surfaces.

**Figure 2:**
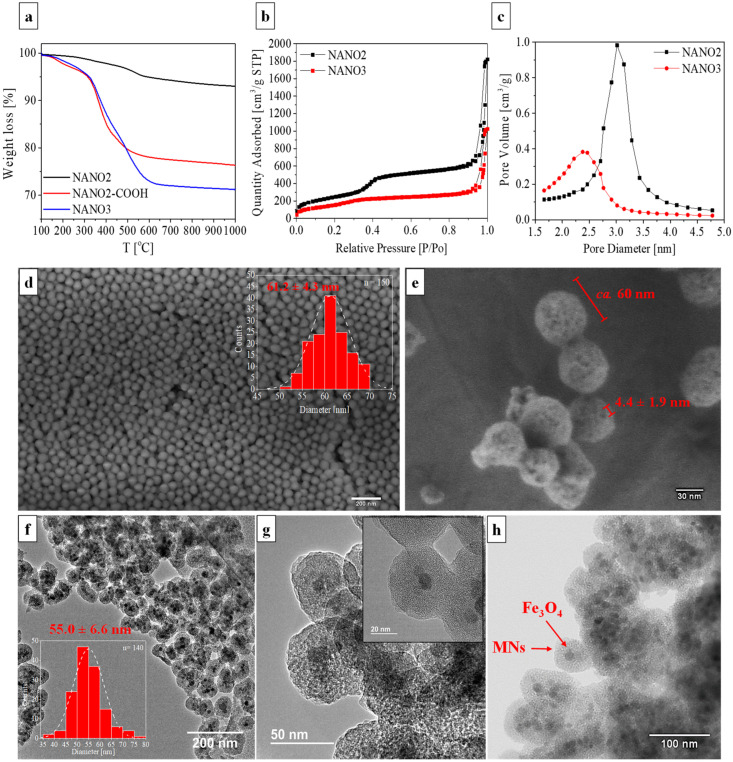
TGA weight loss curves for NANO2, NANO2-COOH and NANO3 (m_i_ = 5.0 mg) (**a**). N_2_-isotherms (**b**) and pore size distribution (**c**) of NANO2 and NANO3. SEM micrograph of NANO2-COOH, inset: particle size distribution (**d**) and HRFESEM picture of NANO3, with distinguishable spherical shape and porosity (**e**). TEM images of NANO3, inset: particle size distribution (**f**,**g**) and brightfield STEM image of NANO3 (**h**).

The presence of the very same aliphatic chains was evident in TGA, with NANO2-COOH and NANO3 having weight losses of ca. 20% and 30%, respectively (Figure 2a), where the 10% difference between them corresponded to the 3-aminopropyl groups at the surface of SiQDs. Again, this confirmed the successful functionalization of NANO2 surface towards NANO3. The mesoporosity of the systems, before and after surface modifications, was verified by the N_2_ adsorption–desorption type IV isotherms of NANO2 and NANO3, both showing a typical, accentuated adsorption step gradient at P/P0 of ca. 0.35–0.40 (Figure 2b). Surface area, porous volume and pore diameter were assessed by applying Brunauer Emmett and Teller (BET) and Barrett-Joyner-Halenda (BJH) methods, with NANO2 having 912,43 m^2^/g, 0.862 cm^3^/g and 30.1 Å, and NANO3 having 580.33 m^2^/g, 0.443 cm^3^/g and 23.8 Å, respectively (Figure 2c,d). The decrease in porosity and pore diameter was indicative of efficient surface functionalization, where the aliphatic chains and SiQDs partially capped the pores of the silica mesoporous matrix. Notwithstanding, the high porosity of the systems confirmed their availability as cargo delivery vectors, permitting the loading of drugs of interest.

The morphology and size of NANO2-COOH and NANO3 were confirmed by SEM and TEM analyses. Both systems had an unquestionable spherical shape, as depicted in Figure 2d,e, and an average size of about 60 nm. Spherical particles and sizes between 1–100 nm have been repeatedly reported to be responsible for longer circulation times, higher internalization, stability, and interaction with cellular walls, which are all necessary for biological applications and effective drug delivery [51], specifically to target in-body, tumor-associated bacterial infections. Moreover, the porosity of the system was also evidently depicted in the HRFESEM picture of NANO3 (Figure 2e).

From TEM (Figure 2f,g) and brightfield STEM (Figure 2h) images, and as hinted at by the above characterization techniques, it could be concluded that the herein synthesized systems were mainly comprised of single iron oxide cores, successfully functionalized with a silica mesoporous matrix of well-defined limits. Due to their small size and similar nature to the silica mesoporous layer, no SiQDs could be directly distinguished, being only perceived as the loss of the smooth surface of the particles in detriment of an embossed surface with blob-like structures of ca. 3.6 nm (Figure 2g) [42]. Elemental analysis on NANO3 was assessed via dark-field STEM analysis, that confirmed Si, O, N and Fe as the main constituents (Figure S5), with iron atoms being confined to the core of the particles and the remaining elements to the outer silica layer. The assessment of NANO3 degradability under simulated body conditions (Figure S6), demonstrated the complete loss of structure and segregation of NANO3 constituents after just one month of exposure. This loss of structure was evident after just half a month of exposure with only a few NANO3 particles maintaining their integrity among the general aggregation, due to silica dissolution and reorganization. After one month there was a total segregation of NANO3 constituents with the magnetic NANO1 cores found preferably in larger aggregates, free of silica. The total degradation of NANO3 was evident by the inexistence of core-shell spherical particles of NANO3 and the exposed NANO1 cores, being then more exposed to in-body clearance mechanisms.

**Figure 3:**
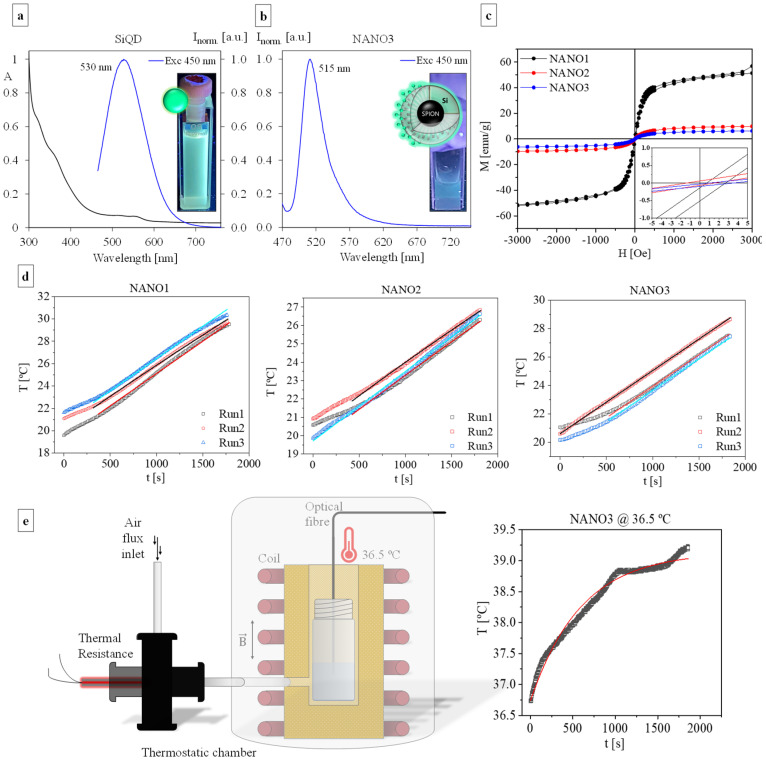
Absorbance and fluorescence spectra of SiQD, after excitation at 450 nm; inset: in-solution picture of SiQD dispersion under 365 nm irradiation (**a**). Fluorescence spectra of as-synthesized NANO3; inset: in-solution NANO3 under 365 nm irradiation (**b**). VSM magnetization loops of NANO1, NANO2 and NANO3 vs. applied field at 273.15 K; inset: close-up of the same charts for hysteresis visualization (**c**). Hyperthermia (heat generation) capacity of NANO1, NANO2 and NANO3 in water, under a 274 kHz and 14 kA/m external AMF, for 1800 s, (environment at 19 °C) (**d**). Controlled temperature hyperthermia set-up and NANO3 hyperthermia heating profile with environmental temperature at 36.5 °C (**e**).

Beyond their physiochemical properties, and as NANO3 synthesis sought to produce hybrid magneto-fluorescent materials, the assessment of their spectral properties (i.e., fluorescence and magnetism) was also of utter importance. As-synthesized aqueous SiQDs, as previously reported [42], due to their incorporation of N and O atoms, have a typical green emission, with a maximum emission band centered at 530 nm (Figure 3a). The anchoring of SiQDs on the surface of NANO2-COOH rendered the final NANO3 with a similar, although slightly blue shifted, bluish-green, emission, with an emission maximum centered at 515 nm (Figure 3b). This blue shift, as hinted at in previous reports [36,52], arose from the change in SiQD surface groups, as part of the original -NH_2_ moieties changed into amide > NH groups during the cross-linking with NANO2-COOH, being responsible for the bluish tonality. Additionally, this also indicated that the final fluorescence was not affected by the presence of a magnetic core, with the spacing materials (i.e., silica shells and aliphatic chains) effectively protecting the former from the latter.

The retainment of the original NANO1 paramagnetic properties through all synthetic steps towards NANO3, was also confirmed by the VSM analysis of magnetization loops of NANO1, NANO2, NANO3 (Figure 3c). While bare NANO1 (in chloroform) showed a high saturation magnetization of 56.8 emu/g, typical of small SPIONs [53], for NANO2 and NANO3 the increase in their non-magnetic particulate masses was accompanied by a significant decrease in saturation magnetization to 9.8 and 6.2 emu/g, respectively. The lack of significant hysteresis in the magnetization loops of all particles corroborated the above statements on the single domain crystallinity of the NANO1 lattice. Moreover, the propagation of this pattern to NANO2 and NANO3 showed that, despite all modifications, both the core lattice single domain and its capacity to instantly orient and magnetize itself to an external field remain unchanged. This is also pointed out by their near zero coercivity, with only NANO1 having a small coercivity of 3 Oe. The decrease in both magnetization and coercivity have been attributed to surface spin effects and changes in the anisotropy constant after modifications [54].

These results were reflected in the hyperthermia profiles of all NANO1, NANO2 and NANO3, with all three being capable of efficiently generating heat and inducing temperature rises of ca. 10 °C when submitted to a 274 kHz AMF and 2280 W, for 1800 s (Figure 3d). The overall heating for each sample showed a linear relationship with time and corresponded to approximately similar power losses of 0.0367, 0.0588 and 0.0503 W for NANO1, NANO2 and NANO3, that were used, along with their masses, to estimate each sample SLP [55]. NANO2 (4.0 mg) and NANO3 (4.0 mg) showed SLPs of 11 ± 1 and 13 ± 3 W/g_NANO_, respectively, that had no significance, since a large fraction of their mass came from their non-magnetic silica shells. The magnetic cores in these samples were similar to the ones in the sample NANO1 (0.22 mg) and were characterized by an SLP of 170 ± 17 W/g_NANO_ (235 ± 24 W/g_Fe_), typical of small SPIONs with diameters between 11–14 nm and suitable for hyperthermia applications [54,56]. This result, allied to the lack of hysteresis losses and a particle size of about 12 nm, indicated that the heating of the NANO1 (cores) arose from Neel and Brownian relaxations, with a predominance of the former [57,58]. NANO2 and NANO3 could also have had an increased contribution of Brownian relaxation, due to the enlargement of the coated nanoparticles. Normalizing the SLPs values for NANO2 and NANO3 by the mass of iron, the values obtained were 270 ± 35 and 295 ± 47 W/g_Fe_, respectively, in agreement with the value of NANO1. Thus, the hypothesis that both these systems could also be used for hyperthermia was defended. To mimic an in-body application, NANO3 hyperthermia heating capacity was tested under a controlled temperature environment of 36.5 ± 1.0 °C (Figure 3e). The assay yielded similar results to those at room temperature, with the system having the capacity to increase the medium temperature up to 39.5 °C, and having an overall power loss of 0.0577 W, corresponding to an SLP value of 14 ± 2 W/g_NANO_ (339 ± 52 W/g_Fe_). The maximum temperature reached, although below that usually used in therapeutic hyperthermia approaches, has been demonstrated to be sufficient to not only induce mild cell wall disturbances, biofilm detachment and immune system stimulation (inducing febrile temperatures) [59], but also to increase drug delivery, without their own deactivation [60,61,62].

With these results, we could state that we were able, for the first time, to successfully synthesize hybrid magneto-fluorescent nanoparticles for drug delivery, comprising sustainable, biocompatible and biodegradable materials of iron oxide for the magnetic core, silicon for the fluorescent SiQD probes and silica for the mesoporous matrix.

### 2.2. Loading, pH-Mediated Release and Hyperthermia Release Assays

To render the synthesized nanocarriers with antimicrobial properties, single and loaded combinatory formulations of OFLO and DOX were physically adsorbed into the NANO2-COOH empty mesoporous matrix, in PBS 0.01 M (pH 7.4). The efficient pore loading was confirmed by DLS, with NANO2@D, NANO2@O and NANO2@DO showing more negative ζs (−33.0 ± 0.7 mV, −35.9 ± 0.4 mV, −35.6 ± 2.7 mV respectively), pointing towards a negligible binding interaction with surface carboxyl groups and a preference for the empty pores. This also translated into smaller HD (126.1 ± 8.8 nm, 121.8 ± 1.4 nm and 148.7 ± 22.0 nm, respectively) and higher stability (PDI of 0.155 ± 0.013, 0.207 ± 0.004 and 0.275 ± 0.033, respectively), with the slight positive drugs within the pores inducing a higher polarization of the surface.

**Figure 4:**
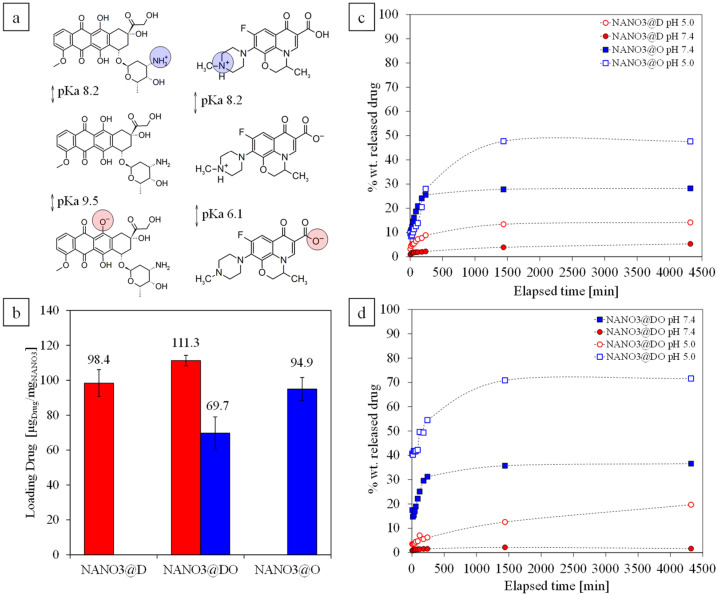
Ionization of DOX and OFLO in aqueous solutions (**a**). NANO3 systems loading capacities of DOX and OFLO (n = 5), in single and dual combinatory formulations (**b**). DOX (circles) and OFLO (squares) release profiles from NANO3@D and NANO3@O (**c**) as well from NANO3@DO and (**d**) under physiological and acidic conditions (n = 3).

The drug content of the loaded systems was evaluated at each surface modification step, rendering the final NANO3@D with 98.4 ± 7.7 µg_DOX_/mg_NANO3_, NANO3@DO with 111.3 ± 3.0 µg_DOX_/mg_NANO3_ and 69.7 ± 9.3 µg_OFLO_/mg_NANO3_, and NANO3@O with 94.9 ± 6.7 µg_OFLO_/mg_NANO3_ (Figure 4). The high encapsulation capacities were in accordance with those reported in other studies for similar mesoporous systems [63], with OFLO successfully replicating the results obtained in other works of our group [28,33]. These attributed it to the correct selection of the loading media’s pH (pH7.4) during NANO2-COOH loading, with both DOX (pKa-_NH2_ = 8.2, pKa_phenol_ = 9.5) and OFLO (pKa-_COOH_ = 6.1, pKa_(piperizinyl ring)_ = 8.2) being partly positively charged at physiological pH (Figure 4a) [64,65,66,67,68] and having preferential, although not permanent, attractive electrostatic interaction with NANO2-COOH, whose surface carboxylic groups and pore silanol groups rendered it with a net negative charge. Regarding the combinatory drug formulation, the relative reduced DOX solubility in the buffered media contributed to the preferential loading of DOX within the pores of NANO systems (Figure 4b), in detriment of OFLO encapsulation [69].

Interestingly, the fluorescent profiles of the loaded NANO3 systems (Figure S7) also reflected the incorporation of both OFLO and DOX, by having their respective 450 nm and 590 nm emission peaks alongside that of grafted SiQDs (λ = 515 nm), after excitation at 330 nm, 450 nm and 480 nm.

Release assays were performed over the final NANO3 loaded systems, in both physiological (pH 7.4) and acidic (pH 5.0) conditions (Figure 4c,d) for 72 h, to simulate a pH-responsive drug delivery of each drug and facilitate an understanding of its drug efficiency enhancement and toxic side effect inhibitions in a future dual application, when targeting cancer and associated opportunistic bacterial infections. The DOX delivery capacities, both from NANO3@D and NANO3@DO, were higher at pH 5.0 (i.e., of 14.2 % wt. and 19.7 % wt., respectively) than at pH 7.4 (i.e., of ca. 5.0 % wt. and ca. 3.0 % wt., respectively), which was in accordance with the already reported tendency for aminated DOX-containing delivery nanocarriers [34,42], as is the case of all NANO3 systems. Here, there was a compromise between DOX solubility at different pHs and the surface steric hinderance created by the aminated SiQD, with acidic pHs not only increasing DOX solubility in aqueous media, but also the protonation of surface NH_2_ groups and the creation an electrostatic repulsion between surface SiQDs. A spectroscopic analysis of SiQDs in both pHs confirmed this protonation phenomena, with SiQD shifting their emission from green to blue when lowering medium pH from 12 to 4.0 (Figure S8). This last phenomenon left the pores open enough to release entrapped DOX molecules, even if slowly, due to the partly positively charged DOX [70]. It should also be noted that at pH 5.0, DOX delivery from NANO3@DO was more time-paced, contrary to the initial burst release from NANO3@D at the same pH, which might have been due to the simultaneous presence of positively charged OFLO molecules.

Similarly, OFLO delivery was also shown to be highly dependent on pH variations, nearly doubling its maximum release from ca. 30 % wt. to 47.6 % wt. and 71.6 % wt. for NANO3@O and NANO3@DO, respectively, when decreasing the pH from 7.4 and 5.0. The increased release of OFLO from NANO3@DO at pH 5.0, when compared to that of NANO3@O, was accompanied by the highest delivery of DOX. Again, this suggested that, when in a combinatory formulation, both positively charged OFLO and DOX catalyzed each other’s delivery, through their increased electrostatic repulsive interaction within the NANO3 systems’ pores.

From a mechanistic point of view, by fitting the collected data to the semi-empirical Korsmeyer–Peppas model, and its further linearization (Equation (1)) for the determination of both *n* and *K_m_* parameters, we could conclude that, for all loaded NANO3, delivery was ruled by quasi-Fickian diffusion mechanisms (Table S2). This was given by an *n* < 0.43, for spherical particles, which indicated there was no interference from matrix swelling or erosion phenomena in the diffusion of drugs [71].

(1) $$\ln\frac{M_{t}}{M_{\infty }}=\ln K_{m}+n\cdot \ln t$$

Briefly, this preference for acidic media makes NANO3 optimal to actuate in cancer environments where pH is generally lower, releasing both anticancer and antibiotic properties in higher quantities and, thus, addressing both cancer growth and opportunistic bacterial infections.

**Figure 5:**
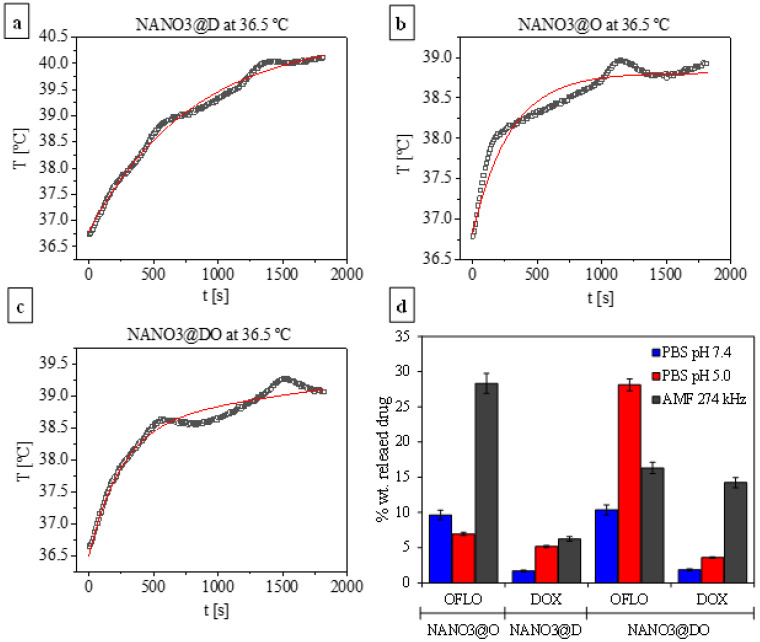
Hyperthermia heating profiles of NANO3@D (**a**), NANO3@O (**b**) and NANO3@DO (**c**), at 37 °C, under an AMF at 274 kHz and 2880 W, for 1800 s (30 min); black lines: experimental data, red lines: fitting of the experimental data. Maximum released drug from NANO3@D, NANO3@O and NANO3@DO, at 37 °C, under different conditions (*n* = 3): blue = 0.01 M PBS pH 7.4, red = 0.01 M PBS pH 5.0 and grey = AMF at 274 kHz (**d**).

The incorporation of the magnetic NANO1 core within the structure of NANO3 renders it with the capacity to not only generate heat under an alternating external magnetic field through synchronous vibration, but also, as previously reported, to increase drug delivery and therapeutics performance [72,73]. Thus, the use of NANO3 systems’ magnetic core to promote a hyperthermia-mediated delivery was also studied for each drug formulation. For this, the resuspended NANO3@D, NANO3@O and NANO3@DO, in a concentration of 2 mg/mL, were submitted to an external AMF of 274 kHz and 2880 W, for 1800 s (30 min) at 37 °C. The analysis of the obtained hyperthermia heating profiles showed that all could heat the media to final temperatures of 39.0 °C (NANO3@O and NANO3@DO) and 40 °C (NANO3@D) (Figure 5a–c). Moreover, while DOX encapsulation led to no changes in NANO3@D hyperthermia capacity, with an SLP of 14 W/g_NANO_ (322 W/g_Fe_), similar to that of empty NANO3, the presence of OFLO in NANO3@O and NANO3@DO produces higher SLPs of 21 and 24 W/g_NANO_ (506 and 557 W/g_Fe_). This increase for OFLO-containing formulations was due to the observable increased viscosity of the suspensions with OFLO, which was directly related with Brownian relaxation times and, thus, with increases in SLPs [54].

Regarding the release of the encapsulated drugs, the obtained released amounts were compared with those obtained from the releases in PBS at pH 7.4 and pH 5.0, for the same time point (i.e., 30 min). As depicted in Figure 5d, the use of hyperthermia as stimuli significantly boosted the release of OFLO and DOX, when compared to the release amounts of each at pH 7.4 and 5.0. This was evident for NANO3@O, where the release of OFLO under AMF influence was 3 times more than at both tested pHs, for NANO3@D, where the release of DOX was ca. 3.5 times more than at physiologic pH, and, lastly, for NANO3@DO, where the release of OFLO and DOX under AMF was 5% more than that at pH 7.4 and 3 times more than any of the tested pH levels, respectively. Remarkably, the release profiles after this short time were comparable to those obtained after 3 to 4 h of incubation at pH release, and, thus, supported the idea that AMF employment boosts drugs release by inducing extra particulate vibrations, local heat and higher permeability [74,75]. As in the pH release assays, for AMF hyperthermia assays DOX release was higher when in a dual formulation with OFLO, again supporting the idea that both catalyze each other’s delivery, through their increased electrostatic repulsive interactions within the NANO3 systems’ pores.

In general, the cumulative OFLO release from either single or dual combinatory formulations was more intense than that of DOX, regardless of the selected pH condition or whether under the influence of an external AMF or not. From the obtained results it could be concluded that in a possible combinatory treatment, there was an initial burst release of OFLO into the medium, regardless of the pH, killing most local opportunistic bacteria, followed by a paced and controlled release of DOX to treat the associated cancer cells.

### 2.3. Antimicrobial Activity

The potential use of the produced nanoparticles as antimicrobial materials against cancer-related, opportunistic bacterial strains was finally tested for a battery of five standard strains: *E. coli* [76], *S. aureus* [77], MRSA [78], *P. aeruginosa* [79] and *E. faecalis* [80]. These standard strains were selected due to their identified relationship, and locality, to some types of tumors, as well as their known response to the selected drugs [28]. With the assay, we aimed to determine the minimum inhibitory concentration of NANO3, NANO3@O, NANO3@D and NANO3@DO, perceived as the lowest concentrations at which bacterial growth was ≤50% (MIC_50_) and ≤10% (MIC_90_) [81]. All samples were resuspended in water and diluted in growth medium.

**Figure 6:**
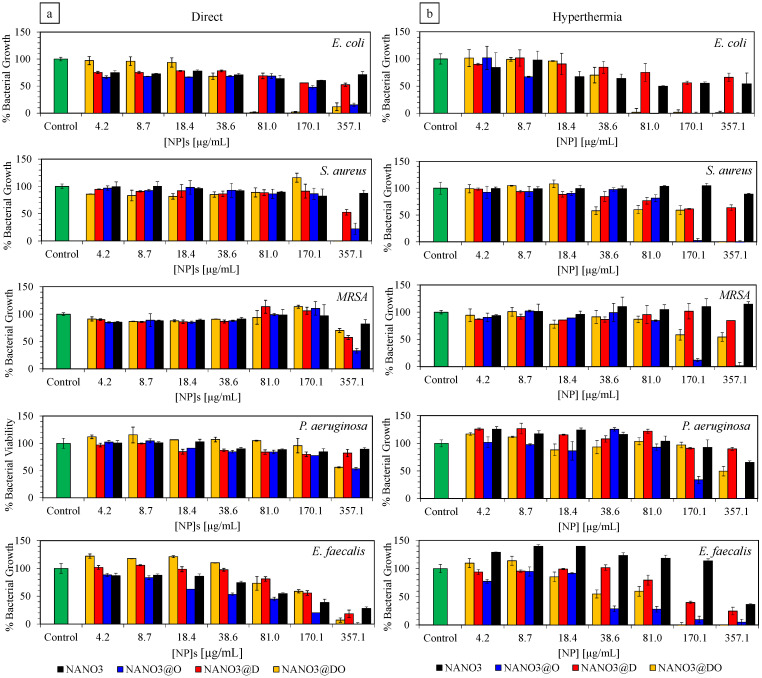
Inhibitory activity, as bacterial growth (%), of NANO3, NANO3@O, NANO3@D and NANO3@DO against (*from top to bottom*) *E. coli*, *S. aureus*, MRSA, *P. aeruginosa* and *E. faecalis* strains, by direct application (**a**) and after-hyperthermia (AMF of 274 kHz) application (**b**). Concentrations given as µg of nanoparticle per mL.

As is shown in Figure 6a, for the direct application of suspensions, the inhibitory activity of the NANO3 systems against most strains arose from the loaded drugs, specifically ofloxacin. This was exempted only by *E. faecalis*, where there was significant inhibitory activity from the nanoparticles at the higher concentrations, with only NANO3@O having a slightly higher activity than NANO3. Similar to other reported assessments [27,28], *E.coli* was the most susceptible strain to this combination of drugs and, whereas a distinguishable MIC_50_ and MIC_90_ were registered for NANO3@O, at 170.1 and 375.1 µg/mL, for NANO3@DO a stronger inhibitory activity was perceived as the overlap of both MIC_50_ and MIC_90_ at 81 µg/mL. Their actions against *S. aureus*, MRSA and *P. aeruginosa* were, however, reduced when compared with that seen for *E. coli*. For *S.aureus*, NANO3@O and NANO3@DO only showed, respectively, MIC_50_ and MIC_90_ at the highest concentration of 357.1 µg/mL. Conversely, for MRSA only NANO3@O kept its MIC_50_ at 357.1 µg/mL, with NANO3@DO no longer having the same activity and only being capable of reducing bacterial growth by 30%. Lastly, against *P. aeruginosa*, NANO3@O and NANO3@DO both showed MIC_50_ at the highest concentration of 357.1 µg/mL. It should be noted, that this small activity against *P. aeruginosa* might have arisen from an increased biofilm production, due to the presence of Fe species from the core of NANO3 systems, as all samples had a strong green coloration and high viscosity after the assay.

The use of hyperthermia before the application of nanoparticles has been confirmed to increase the release of the loaded drugs, and this was also reflected in the antimicrobial activity of the NANO3 systems (Figure 6b). In *E. coli*, the determined MIC_50_ and MIC_90_ of NANO3@O were reduced to a particle concentration of 18.4 µg/mL. This approximately 90% reduction in the inhibitory concentration was supported by the higher OFLO release after the application of AMF and, thus, higher availability in the medium. The use of an AMF also benefited the antimicrobial activity of NANO3@O and NANO3@DO against *S. aureus* and MRSA, reducing the first’s MIC_90_ to 170.1 µg/mL, for both bacteria, and the second’s MIC_50_ to 38.6 µg/mL and 170.1 µg/mL, against *S. aureus* and MRSA, respectively. Similarly, against *P. aeruginosa*, NANO3@O saw its MIC_50_ reduced by half to a concentration of 170.1 µg/mL after the application of an AMF. Lastly, regarding *E. faecalis* susceptibility to NANO3@O and NANO3@DO, after AMF-induced hyperthermia, the MIC_50_ of both NANO3@O and NANO3@DO were reduced by a 4.5 factor to a concentration of 38.6 µg/mL. Interestingly, in this case, the inhibitory activity of these two loaded systems no longer followed the tendency of the empty NANO3, that only had its MIC_50_ at 357.1 µg/mL. The antimicrobial activity of NANO3@D was similar to that of empty NANO3, as expected, showing only some activity against *E. faecalis*

The obtained results were in accordance with the release profiles after AMF hyperthermia, where there was OFLO delivery, especially in its single formulation. Overall, we could conclude that upon application of an external AMF, NANO3@O and NANO3@DO drug delivery capacity and antimicrobial activity were enhanced, promoting the dispensing of both antibiotic and anticancer drugs in a future combinatory AMF-mediated therapy to tackle opportunistic cancer-related bacterial infections.

## 3. Materials and Methods

### 3.1. Chemicals

Iron(III) chloride hexahydrate (FeCl_3_.6H_2_O, 97.0−102.0%), iron(II) chloride hydrate (FeCl_2_.H_2_O, 99%), ammonium nitrate (NH_4_NO_3_, 99.999%), N-hydroxysuccinimide (NHS, 98+%), ethylenediaminetetraacetic acid tetrasodium salt hydrate (EDTA, 98%) and tetraethyl orthosilicate (TEOS, ≥99.999% metals basis) were bought at AlfaAesar. The *n*-Cetyltrimethylammonium bromide (CTAB), (3-aminopropyl) triethoxysilane (APTES, ≥98%), thioglycolic acid (≥99.9%), succinic anhydride (≥99% GC), (+)-sodium L-ascorbate (crystalline, ≥98%), N-3-dimethylaminopropyl-n-ethylcarbodiimide hydrochloride (EDC, ≥97%), acetone (≥99.5% GC), 4-morpholineethanesulfonic acid (MES, ≥99%), TRIS (hydroxymethyl)aminomethane hydrochloride (TRIS HCl) and phosphate-buffered saline (PBS, tablets) were purchased from Sigma Aldrich. Ethanol absolute (EtOH, extra pure), hydrochloric acid (HCl, 37%) and sodium hydroxide (NaOH) were bought at Scharlab, SL. Oleic acid (OA, 65.0–88.0 %) was acquired from Honeywell Fluka and sodium chloride (NaCl, 99.0–100.5%) at PanReac AppliChem. Bacto^TM^ Glycerol was bought from Becton Dickinson&Co (Sparks, MD, USA). Muller Hinton Broth (MHB) and Trypto Casein-Soy Broth (TSB) were obtained from Biokar Diagnostics. All reagents were used as acquired, without any further purification, and all solutions, unless otherwise indicated, were prepared with deionized Millipore miliQ water.

### 3.2. Instrumentation

Temperature controlled incubations and release studies were performed in a HC24 PCMT Thermo-shaker (Grant Instruments, Cambridge, UK). Magnetization studies were performed in a Vibrating Sample Magnetometer (VSM) Lakeshore 7304 (Lake Shore Cryotronics, Westerville, OH, USA), with a maximum applied magnetic field of 1.45 T. HRFESEM (and EDS analysis) and SEM images were taken with a GeminiSEM 500 Fiel Emission Scanning Electron Microscope (ZEISS Oxford Instruments) and a FEI Helios NanoLab 450S DualBeam—FIB with UHREM FEG-SEM. TEM images and EDS analysis were acquired in a 200 kV JEM 2100F JEOL–GATAN Oxford Nanomegas, with X-Ray Detector (EDX) DIGISTAR/ADT 3D/ASTAR, from Universitat Politècnica de València (UPV). DLS experiments were performed in a Malvern Nano ZS Zetasizer (633 nm laser diode). UV–Vis absorption spectra were obtained in a Jasco V-650 spectrophotometer and fluorescence spectroscopy measurements were taken in a HORIBA Scientific FLUOROMAX-4 spectrofluorometer, from the Bioscope Group LAQV- FCT NOVA, and a JASCO FP-8300 spectrophotometer, from UPV. Powder X-ray diffraction (PXRD) measurements were taken on a D8 Advance diffractometer (Bruker, Billerica, MA, USA). Thermogravimetric analyses were carried out on a TGA/SDTA 851e balance (Mettler Toledo, Columbus, Ohio, USA) in an oxidizing atmosphere (air, 80 mL min^−1^). Inductively coupled plasma atomic emission spectroscopy (ICP-AES) was performed in an ICP Ultima model (Horiba Jobin-Yvon, Longjumeau, France). N_2_ adsorption–desorption isotherms were recorded with a Tristar II Plus automated analyzer (Micromeritics, Norcross, GA, USA). The samples were degassed at 90 or 120 °C under vacuum overnight. ATR-infrared spectra were collected in a Tensor 27 (Bruker, Billerica, MA, USA), and ^1^H/^13^C nuclear magnetic resonance spectroscopy (NMR) performed in a Bruker FT-NMR Avance 400 (Ettlingen, Germany) spectrometer.

Hyperthermia studies were conducted under an AC magnetic field, obtained with a water cooled two-spire Helmholtz coil, operated by an Ambrell EASYHeat 0224 power generator. Samples were placed in a thermostatic chamber with temperature controlled by the flow of heated air between the sample container and the cork-insulator that isolated the system from the coils. The whole ensemble was isolated from external air convection by an acrylic chamber.

Transparent flat-bottom sterile 96-well plates were purchased from Greiner Bio-One and U-bottom opaque black 96-well plates from RatioLab (Dreieich-Buchschlag, Dreieich, Germany). Aseptic bacterial assays and modifications were handled in a Steril-VBH laminar flux chamber. Turbidity for each assayed bacterial suspension was adjusted in a DEN-1B McFarland Densiometer (Grant-Bio, Cambridge, United Kingdom). Incubations were conducted in a Mermmet Incubator B10, at 37 °C. Optical density (OD, 600 nm) measurements were conducted in a UV-Vis CLARIOstar^®^ Plus spectrophotometer (BMG Labtech, Ortenberg, Germany).

### 3.3. Superparamagnetic Iron Oxide Nanoparticles Synthesis—NANO1

The synthesis of well dispersed superparamagnetic iron oxide (Fe_3_O_4_) nanoparticles (NANO1) was achieved by a modification of the co-precipitation approach detailed by Zhang et al. [43]. Briefly, 2.4 g of FeCl_3_.6H_2_O and 1.0 g of FeCl_2_.H_2_O were stirred until total dissolution in 10 mL of miliQ H_2_O, at 80 °C, under continuous Ar bubbling. An amount of 5 mL of NH_4_OH (25 wt.%) was then added, and the resulting black solution stirred for an additional 1 h, at 80 °C under continuous Argon (Ar) bubbling. After this, 425 µL of oleic acid were injected into the mixture, which was bubbled for an additional 5 min and then sealed in Ar atm, and stirred for 1.5 h at 80 °C. The OA-coated NANO1 were magnetically decanted with the aid of a neodymium magnet, washed 10 times with miliQ H_2_O, to remove OA excess, and transferred to 20 mL of chloroform. NANO1 concentration was estimated through the total content of Fe, determined by ICP-AES; the size of NANO1, determined by TEM; and its crystalline lattice structure [82], yielding ca. 6.8 × 10^15^ particles/mL (2.4 × 10^17^ particles/g).

### 3.4. Mesoporous Silica Shell Surface Growth and Surface Modifications—NANO2

In a typical approach, 150 mg of CTAB were dissolved in 10 mL of miliQ H_2_O, mixed with 0.74 mL of NANO1 and sonicated for 1 h, in an ultrasound bath at 35 KHz and 50 °C. The resulting emulsion was then heated to 70 °C and stirred for 30 min to evaporate the residual chloroform. Then, 30 mL of miliQ H_2_O, 10 mL of ethylene glycol and 0.7 mL of NH_4_OH (25 wt.%), were added, in this order, to the mixture which was stirred at 70 °C. After 30 min of homogenization, 750 µL of TEOS were introduced dropwise to the reaction which was stirred for 3 h at 70 °C. The as-prepared sample, NANO2, was cooled to room temperature, centrifuged, washed in MeOH 3 times and left to dry at 70 °C.

For the modification of NANO2 surfaces, 50 mg of NANO2 were resuspended in 12 mL of miliQ H_2_O and mixed with 1.2 mL of a THF solution containing 350 µL of APTES, for 24 h at room temperature. The resulting NANO2-NH_2_ were collected by centrifugation and washed with H_2_O and acetone, before being resuspended in 5 mL of acetone and mixed with 3 mL of a 1.5 M succinic anhydride solution in acetone. The mix was stirred for another 24 h, at room temperature, and the samples collected by centrifugation and washed in MeOH. CTAB removal from the pores of the resulting NANO2-COOH samples was achieved by resuspension in 20 mL of 3 mg/mL methanolic solution of NH_4_NO_3_, for 1 h at 60 °C (3 times).

### 3.5. Silicon Quantum Dots (SiQDs) Synthesis

In an adaption of the synthesis detailed by Marcelo et al. [42], green emissive silicon quantum dots (SiQDs) were prepared by dissolving 50 mg of sodium ascorbate in 10 mL miliQ H_2_O and mixing with 2 mL of APTES. The reaction was initially stirred for 40 min at 50 °C and aged for 24 h at room temperature.

### 3.6. Magneto-Fluorescent Nanoconjugates—NANO3

Magneto-fluorescent nanoconjugates (NANO3) were obtained by conjugation of SiQDs’ amine terminal moieties and NANO2-COOH, via a two-step EDC/NHS crosslinking reaction in a 1:3:3 COOH:EDC:NHS molecular ratio. Briefly, 20 mg of NANO2-COOH were resuspended in 4 mL MES Buffer 0.1 M (pH 6.0), containing 19.2 mg EDC and 14.4 mg NHS, and stirred for 30 min at room temperature. To the resulting mixture, 4 mL of SiQD were added and stirred for 24 h at room temperature, in the dark. The SiQDs’ basicity was brought to pH 8.0 before conjugation with the modified NANO2-COOH, to ensure optimal reaction conditions. The resulting nanoparticles, NANO3, were collected (10,000 rpm for 10 min) and washed with miliQ H_2_O and MeOH.

### 3.7. Preliminary NANO3 Degradation Study

To simulate body-like conditions and assess the potential degradability of the system, as-synthesized NANO3 were resuspended in 0.01 M PBS pH 7.4 and smoothly agitated (50 rpm) at 37 °C, for a full month. Samples of the exposed suspension were taken at time point zero, after half a month and, finally, after one month, then being visualized by TEM.

### 3.8. Loading and Release Studies of Anticancer-Antimicrobial Drugs

For drug-loaded nanoparticles, as-synthetized NANO2-COOH first had their pores emptied via NH_4_NO_3_ solution route, as detailed above. Ofloxacin (OFLO) and doxorubicin (DOX) were used as antimicrobial and anti-tumoral model drugs and were loaded either alone or conjugated in a 1:1 wt. ratio. A 2 mg/mL doxorubicin stock solution was prepared in miliQ H_2_O and a 2 mg/mL ofloxacin stock solution in PBS 0.01 M (pH 7.0). Loadings were performed in batches of 20 mg of template-free NANO2-COOH. Briefly, 20 mg of NANO2 were resuspended in 3.2 mL of the corresponding drug stock solution and diluted in the same volume of the complementary solvent (Table S1). Co-loading of OFLO and DOX was performed similarly with 20 mg of NANO2-COOH being resuspended in 3.2 mL of OFLO and 3.2 mL of DOX stock solutions.

Each sample was collected by centrifugation (10,000 rpm for 10 min) and washed 4 times with 1 mL of PBS 0.01 M (pH 7.0). All supernatants were isolated and used to estimate, by mass balance, the loading capacity of NANO2-COOH for each drug, via UV-vis spectroscopy at 330 (OFLO) and 480 (DOX) nm. The loaded nanoparticles were then submitted to the same surface modifications described above, yielding NANO3@D, NANO3@O and NANO3@DO and their DOX and OFLO contents were assessed by the same procedure.

Studies on pH-dependent release were conducted in PBS 0.01 M, with pH of 7.4 and pH 5.0, at 37 °C. Briefly, 1 mg of each loaded NANO3 was resuspended in 2 mL of buffer solution and shaken for up to 72 h. After centrifugation (12,000 rpm for 5 min), the released drug content in the supernatant was measured for the 5, 15, 30, 45, 60, 90 min and 2, 3, 4, 24, 72 h time points, by fluorescence: OFLO at pH 7.4 (λ_exc_ = 300 nm, λ_exc_ = 460 nm), OFLO at pH 5.0 (λ_exc_ = 300 nm, λ_exc_ = 490 nm) and DOX (λ_exc_ = 480 nm, λ_exc_ = 560 nm). The drug release mechanism of each loaded NANO3 system was studied assuming the Korsmeyer–Peppas approach (Equation (2)) [71]:

(2) $$M_{t}/M_{\infty }=K_{m}t^{n}$$

where, *M_t_* and *M*_∞_ represent the released and initially loaded masses of drug, *K_m_* for the kinetic constant and *n* the release exponent, from which the type of release was determined.

### 3.9. Magnetic Characterization and Hyperthermia Studies

Magnetization hysteresis loops of NANO1, NANO2 and NANO3 were obtained by vibrating sample magnetometer (VSM), and mass saturation magnetizations and their coercivity calculated accordingly. For that 24.4 mg, 9.5 mg and 13.7 mg of each dry sample, respectively, were submitted to a saturating magnetic field of 1 T.

Induction heating of the nanoparticles was achieved using the experimental set-up (Ambrell based) previously described [26,83], for AC magnetic field with 14 kAm^−1^ amplitude and 274 kHz frequency. NANO1 were prepared in chloroform to yield a final concentration of 5.1 × 10^13^ particles/mL, by dispersing 75 µL of the stock dispersion in a total volume of 3 mL, to equal the estimated number of cores in the tested amounts of NANO2 and NANO3 (4 mg). The two last were dispersed in 2 mL of water (2 mg/mL) prior to testing, sonicated till full homogeneity was achieved and immediately subjected to the alternating magnetic field (AMF). Each assay was run for 1800 s (30 min) and the dispersion temperatures were registered continuously by an optical fiber probe (0.1 °C accuracy).

Specific loss power (SPL) was determined from the measured temperature increase for each sample, assuming a constant dissipated power H for the nanoparticles under ac magnetic field and linear losses to the thermostatic chamber [84]. The SLP was normalized by the magnetic nanoparticles mass, m, according to Equation (3), assuming no physical and chemical changes [55]:

(3) $$SLP=\frac{H}{m}$$

Since NANO2 and NANO3 comprised a non-magnetic layer, normalization was also done per mass of iron.

Magnetic-mediated release was performed alongside hyperthermia studies of drug-loaded NANO3 systems. Similar to that described above, 4 mg of each drug-loaded NANO3 system were resuspended in 2 mL (2 mg/mL), sonicated, heated to ca. 37 °C and placed under an AMF with the same specifications, for 1800 s (30 min), with the thermostatic environment at 36.5 ± 0.5 °C. Each sample was recovered by centrifugation at 10,000 rpm, for 5 min, and their supernatants quantified. Both pellets and supernatants were stored and used in further antimicrobial assays.

### 3.10. Antimicrobial Activity Assessment

The antimicrobial activity of the synthesized nanoparticles was assayed against Gram-negative *Escherichia coli* (ATCC^®^ 8739™) and *Pseudomonas aeruginosa* (ATCC^®^ 9027™), as well as Gram-positive *Staphylococcus aureus* (ATCC^®^ 6538™), methicillin resistant *Staphylococcus aureus* (ATCC^®^ 33591™, MRSA) and *Enterococcus faecalis* (ATCC^®^ 29212™). Bacteria were kept frozen at −70 °C in broth containing glycerol (15% *v*/*v*). An amount of 2.5 mg/mL of the suspensions of all selected nanomaterials (NANO3@D, NANO3@O, NANO3@DO) were prepared in H_2_O and used for all assays. Drug solutions containing the same concentration of the drugs loaded into each material were also prepared, in H_2_O. Bacterial suspensions for all strains were prepared from the subcultured TSA plates, in 3 mL of sterile 0.85% NaCl solution. The turbidity of the suspensions was adjusted to 0.5 on the McFarland scale (*ca.* 10^8^ CFU/mL) and diluted with 0.85% NaCl to about 10^7^ CFU/mL.

To evaluate and quantify the bactericidal and bacteriostatic effects of the tested nanocomposites, all samples were assayed by the broth microdilution method in a 96-well microplate. Briefly, two-fold serial dilutions of each sample, and controls, were prepared in sterile MHB to a final volume of 100 μL per well. Then, each well was inoculated with 10 μL of each previously prepared bacterial suspension (to achieve a concentration of 10^6^ CFU/mL in each well). Drug solutions containing the same concentration of the drugs loaded into each material were used as positive controls. Each material in the absence of bacteria and the bacteria without samples, were used as negative controls for the experiments.

Plates were left to incubate for 18 h, at 37 °C. After the appointed time, an aliquot of each well was subcultured on the surface of TSA plates and incubated for an additional 18 h, at 37 °C, for a naked-eye assessment of the MBC. Magnetic hyperthermia samples were used as above-mentioned, with both pellets and supernatants being mixed and homogenized before being applied to bacteria in the antimicrobial assay.

OD_600_ measurements of the 96-well plates were conducted in a plate reader at 600 nm. Bacterial growth and MIC_50_ were calculated from OD_600_ (Equation (4)):

(4) $$Bacterial\;Growth=\frac{Sample\;OD-NANO\;OD}{Bacteria\;control\;OD}$$

where, *Sample OD* and *NANO OD* stand for the incubated sample OD_600_ signal and the correspondent NANOs suspension OD_600_ control signal.

## 4. Conclusions

Engineered nanomaterials have shown a significant role in the regulation of microbial growth, exhibiting bactericidal and bacteriostatic effects. The regulation of opportunistic bacterial infections during cancer treatments is an important factor in patients’ survival and in the success of therapy. The herein reported nanoparticles seek to address this issue by incorporating multiple parts that render them with simultaneous magnetic and fluorescent properties, as well as cargo delivery capacity of single and combinatory formulations of anticancer and antimicrobial drugs. NANO3 were successfully synthesized with biocompatible and biodegradable materials, comprising a superparamagnetic Fe_3_O_4_ core, a highly mesoporous silica shell and surface fluorescent SiQDs. The system showed a high predisposition to simultaneously incorporate and deliver OFLO and DOX as model anticancer drug and antibiotic, with a preferable release in acidic media, like that of invasive tumors and bacterial infections. The pH dependent release is mediated by surface SiQDs, whose amine terminal groups are sensitive to proton medium concentration and control pore access. Hyperthermia-mediated release and heat generation under an external AMF were confirmed for all NANO3 systems, with all achieving febrile temperatures capable of stimulating the immune system and enhanced drug release. Finally, both NANO3@O and NANO3@DO materials showed effective antimicrobial activity against *E. coli* and *E. faecalis* strains, increased only when submitting them to an external AMF and NANO3@DO against *S. aureus*. AMF application increases the previously detected antibacterial activity, also extending the NANO3 systems’ inhibitory activity to otherwise non-susceptible strains like *S. aureus*, MRSA and *P. aeruginosa*. The successful antimicrobial activity of the system was confirmed, and future studies will seek to apply it in a combined antimicrobial–anticancer therapy, thoroughly screening its cytotoxic and antitumoral activities.

## Data Availability

The data presented in this study are available online within this article or in the Supplementary Materials.

## Supplementary Materials

The following supporting information can be downloaded at: https://www.mdpi.com/article/10.3390/ijms232012287/s1. Reference [85] is cited in the supplementary materials.
