# Supplementary material for: Magneto-Fluorescent Mesoporous Nanocarriers for the Dual-Delivery of Ofloxacin and Doxorubicin to Tackle Opportunistic Bacterial Infections in Colorectal Cancer
Source: Int J Mol Sci. 2022 Oct 14;23(20):12287. doi: 10.3390/ijms232012287 (PMC9603674; doi:10.3390/ijms232012287)
Supplement: Supplementary file 1 [file ijms-23-12287-s001.zip › ijms-1958339-supplementary.pdf]

Magneto-Fluorescent Mesoporous Nanocarriers to Tackle Opportunistic Bacterial Infections in Colorectal Cancer

Gonçalo A. Marcelo [1], Joana Galhano [1], Tiago T. Robalo [2], Maria Margarida Cruz [2], María D. Marcos [3,4,5], Ramón Martínez-Máñez [3,4,5], Maria Paula Duarte [6], José Luis Capelo-Martínez [1,7], Carlos Lodeiro* [1,7], Elisabete Oliveira* [1,7]

1 BIOSCOPE Group, LAQV@REQUIMTE, Chemistry Department, FCT NOVA, Caparica (Portugal)
2 BioISI–Instituto de Biosistemas e Ciências Integrativas,, Faculdade de Ciências, Universidade de Lisboa, 1749-016 Lisboa (Portugal)
3 Instituto Interuniversitario de Investigación de Reconocimiento Molecular y Desarrollo Tecnológico, Universitat Politècnica de València, Universitat de València, Valencia (Spain)
4 Unidad Mixta UPV-CIPF de Investigación en Mecanismos de Enfermedades y Nanomedicina, València, Universitat Politècnica de València, Centro de Investigación Príncipe Felipe, València (Spain)
5 CIBER de Bioingenieria, Biomateriales y Nanomedicina (CIBER-BBN) (Spain)
6 MEtRICs/DCTB, FCT NOVA, Caparica (Portugal)
7 PROTEOMASS Scientific Society, Rua dos Inventores. Madam Parque, Caparica Campus, Caparica (Portugal)

* Correspondence: cle@fct.unl.pt (C.L.E.); ej.oliveira@fct.unl.pt (E.O.)

Tables

Table S1. Reaction conditions for the loading assays of NANO2-COOH with DOX, OFLO and a combination of both.

	m_{NANO2} (mg)	$V_{drug\ stock}$ (mL)	$V_{solvent}$ (mL)	V_{total} (mL)
**OFLO**	20	3.2 [1]	3.2 (miliQ H_2O)	6.4
**DOX**	20	3.2 [2]	3.2 (PBS 0.01M)	6.4
**DOX + OFLO**	20	3.2 + 3.2	-	6.4

[1] OFLO stock (2 mg/mL)prepared in miliQ H_2O
[2] DOX stock (2 mg/mL) prepared in PBS 0.01M (pH 7.0)

Table S2. Korsemeyer-Peppas calculated parameters for all pH related assays.

	pH 7.4				pH 5.0			
	**NANO3@D**	**NANO3@O**	**NANO3@DO**	**NANO3@DO**	**NANO3@D**	**NANO3@O**	**NANO3@DO**	**NANO3@DO**
	DOX	*OFLO*	*DOX*	*OFLO*	*DOX*	*OFLO*	*DOX*	*OFLO*
K_m	0.681	0.043	0.006	0.058	0.007	0.040	0.012	0.071
n	0.25	0.33	0.17	0.30	0.21	0.25	0.32	0.26

Figures

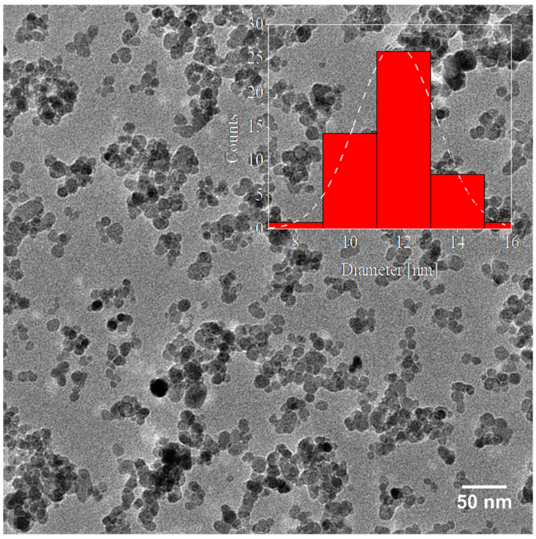

Figure S1. TEM micrograph of as-synthesized NANO1; *inset*: particle size distribution, with d = 11.73 ± 1.47 nm (n = 50). Scale bar of 50 nm.

a

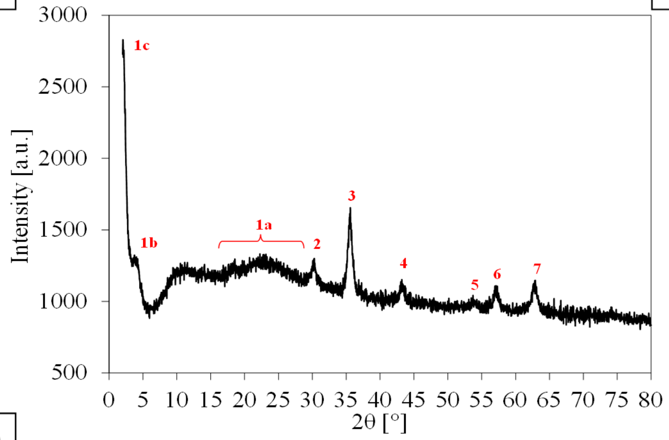

c

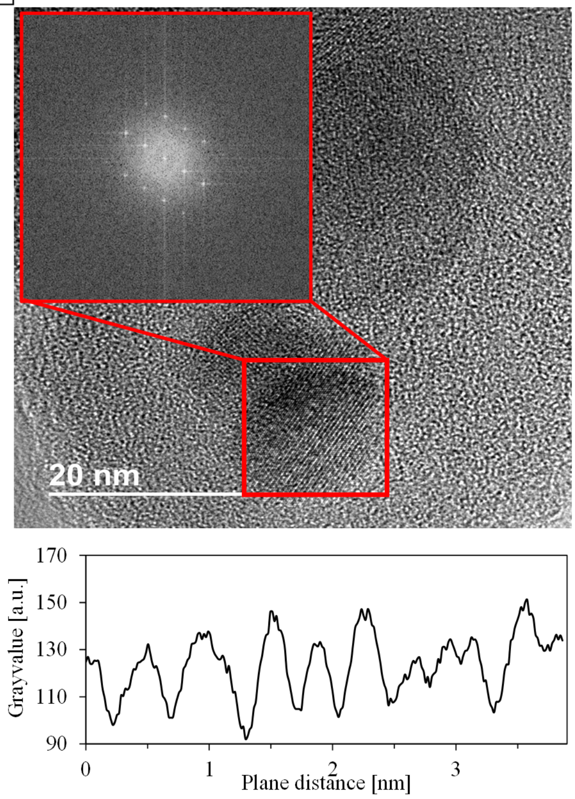

b

Peak	2θ	Intensity	Plane	d_{hkl} (Å)	Material
1a	20-(23)-30	1327	-	-	SiO_2
1b	4.14	1297	<110>	-	
1c	2.16	2076	<110>	-	
2	30.26	891	<220>	2.95	Fe_3O_4
3	35.6	1116	<311>	2.53	
4	43.36	758	<400>	2.08	
5	53.72	-	<422>	-	
6	57.16	720	<511>	1.61	
7	62.56	756	<440>	1.48	

Figure S2. XRD spectrum of NANO2 (**a**), and 2θ peaks' information and attribution to silica (SiO_2) and magnetite (Fe_3O_4) *hkl* lattice distances (**b**). TEM micrograph NANO2 and respective ImageJ generated FTT measurement of the core lattice *d*-spacing of 2.50 Å, assigned to the <311> plane of Fe_3O_4; assessment via grayscale profile plot, by counting the cycles (10) and dividing by total distance (4 nm) [85](**c**).

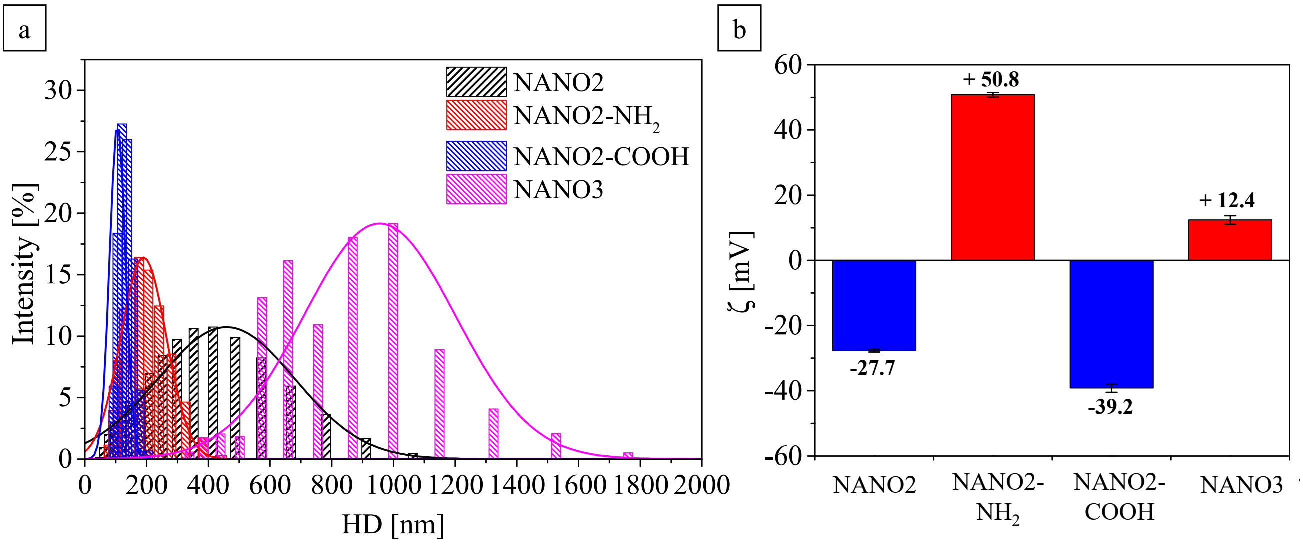

Figure S3. DLS intensity-determined average hydrodynamic diameter (HD) of each synthetic product (a), and correspondent average zeta potential (ζ).

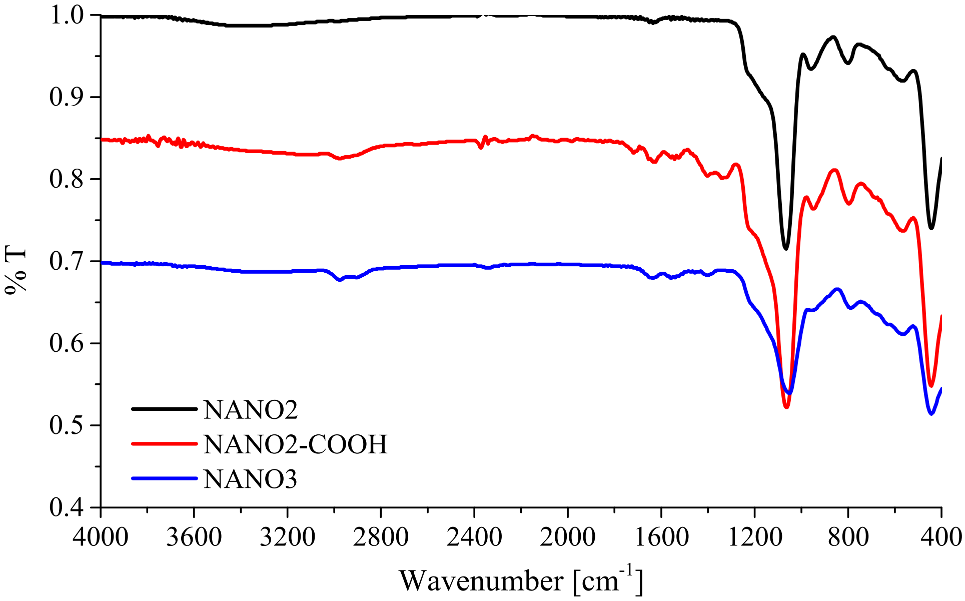

Figure S4. ATR-FTIR spectra of as-synthesized NANO2, NANO2-COOH and NANO3.

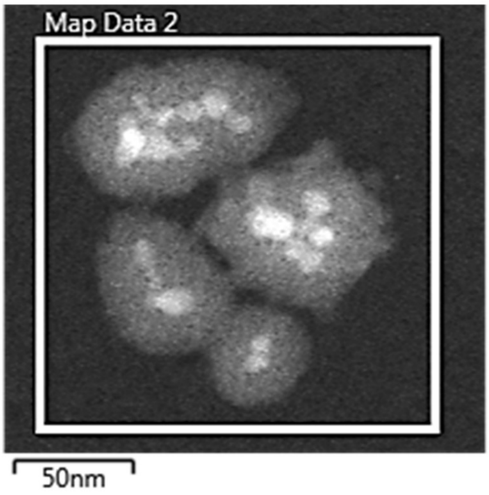

Element	Wt%	Atomic %
**O**	51.3	66.3
**Si**	41.3	28.8
**Fe**	4.7	1.7
**N**	2.7	3.2
Total:	100	100

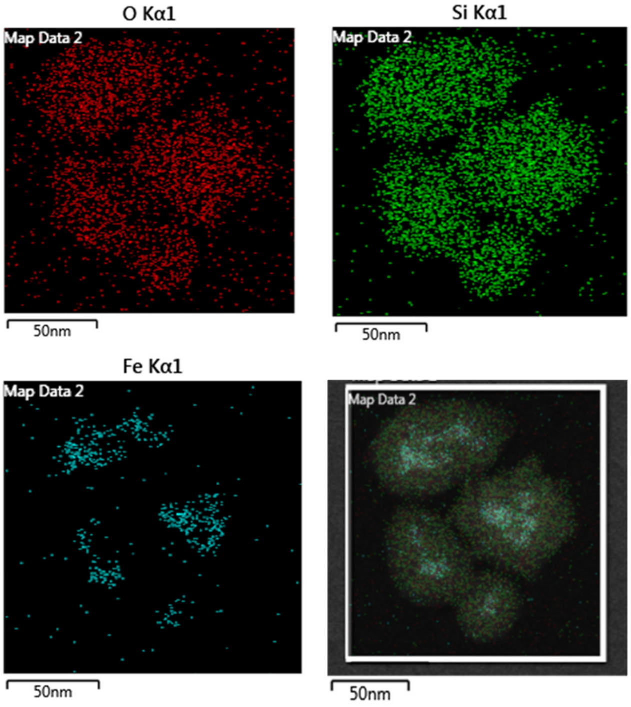

Figure S5. Dark-field STEM image of NANO3 and respective elemental analysis and distribution.

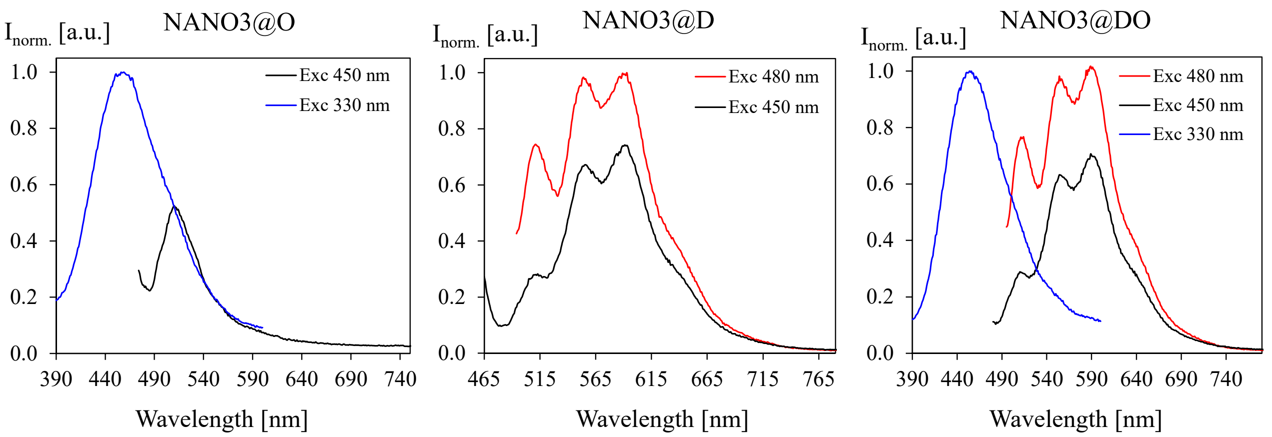

Figure S6. Fluorescence spectra of conjugated SiQD, DOX and OFLO in NANO3@O, NANO3@D and NANO3@DO systems.

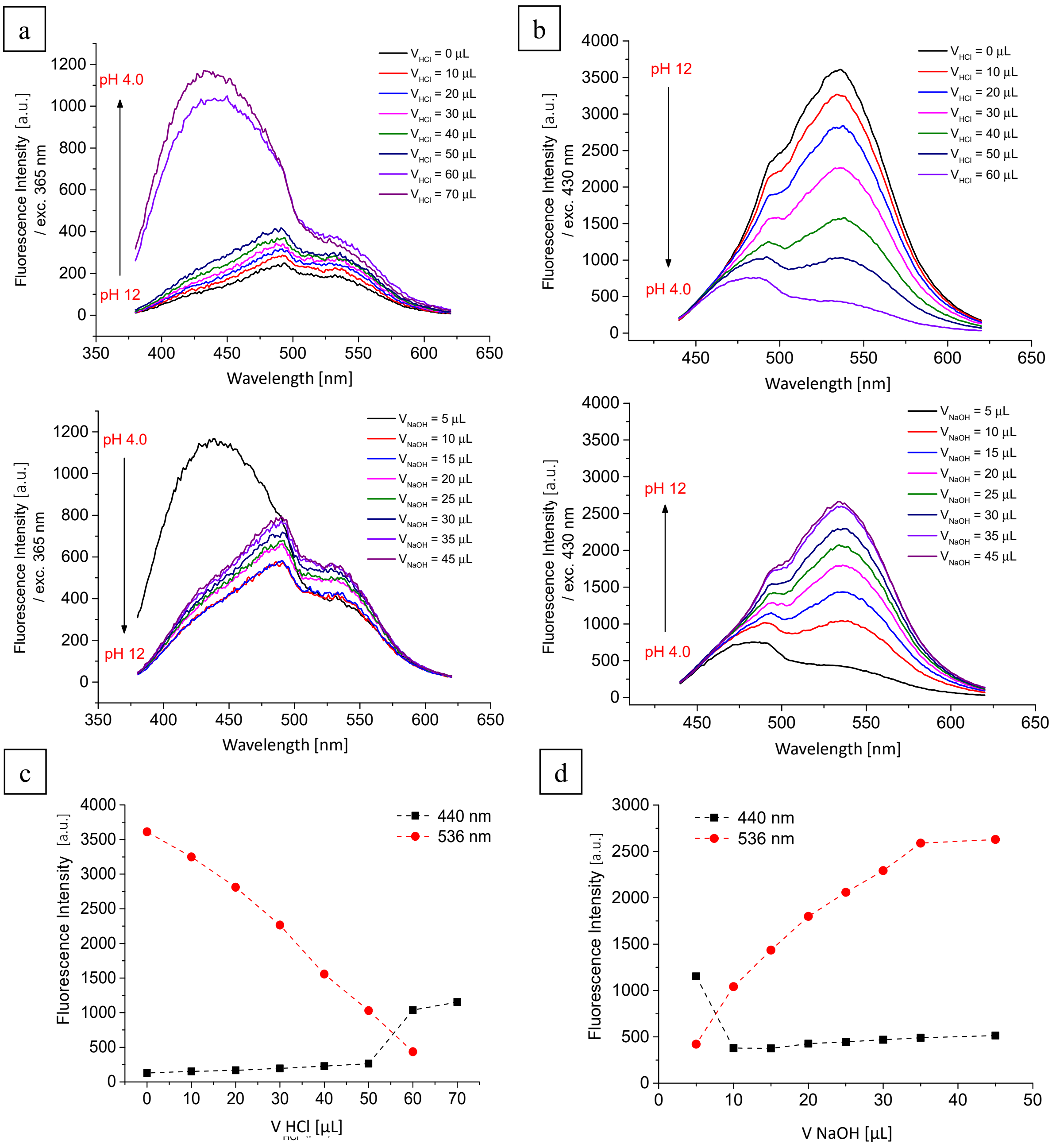

Figure S7. SiQD fluorescence spectra variations by sequential addition of HCl and NaOH 10 and 5 µL, respectively, after excitation at 365 nm (a) and 430 nm (b). Emission maxima (440 and 536 nm) variations with HCl (c) and NaOH (b) added volumes.

85. ImageJ, FFT Measurements, https://imagej.nih.gov/ij/docs/examples/tem/, (accessed 29/03/2022, 2022).
